# Multiemitting Ultralong Phosphorescent Carbonized Polymer Dots via Synergistic Enhancement Structure Design

**DOI:** 10.1002/advs.202400781

**Published:** 2024-03-29

**Authors:** Qipeng Zhang, Shihao Xu, Lanpeng Zhang, Liang Yang, Changlong Jiang

**Affiliations:** ^1^ Institute of Solid State Physics Hefei Institutes of Physical Science Chinese Academy of Sciences Hefei Anhui 230031 China; ^2^ Department of Chemistry University of Science and Technology of China Hefei Anhui 230026 China; ^3^ State Key Laboratory of Transducer Technology Chinese Academy of Sciences Hefei Anhui 230031 China

**Keywords:** carbon dots (CDs), carbonized polymer dots (CPDs), cross‐linking enhance emission (CEE), matrix, room temperature phosphorescence (RTP)

## Abstract

Advancing a metal‐free room temperature phosphorescent (RTP) material that exhibits multicolor emission, remarkable RTP lifetime, and high quantum yield still faces the challenge of achieving intersystem crossing between singly and triplet excited states, as well as the rapid decay of triplet excited states due to nonradiative losses. In this study, a novel strategy is proposed to address these limitations by incorporating o‐phenylenediamine, which generates multiple luminescent centers, and long‐chain polyacrylic acid to synthesize carbonized polymer dots (CPDs). These CPDs are then embedded in a rigid B_2_O_3_ matrix, effectively limiting nonradiative losses through the synergistic effects of polymer cross‐linking and the rigid matrix. The resulting CPD‐based materials exhibit remarkable ultralong phosphorescence in shades of blue and lime green, with a visible lifetime of up to 49 s and a high phosphorescence quantum yield. Simultaneously, this study demonstrates the practical applicability of these excellent material properties in anti‐counterfeiting and information encryption.

## Graphical abstract

Developing room‐temperature phosphorescent carbon dots with extended afterglow and multicolor emission is challenging. This work proposes a novel strategy using o‐phenylenediamine and polyacrylic acid to synthesize carbonized polymer dots embedded in B_2_O_3_ matrix. The resulting materials exhibit dual‐emitting ultralong phosphorescence in blue and lime green shades under different UV excitations, with a remarkable phosphorescence lifetime of up to 49 s and potential applications in anticounterfeiting and information encryption.

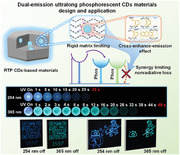

## 1 Introduction

Due to the complexity, high cost, and toxicity associated with conventional room temperature phosphorescent (RTP) materials like pure‐organic phosphorescent materials or metal‐containing inorganic phosphorescent materials, there has been a significant focus on carbon dot‐based RTP materials due to their simple synthesis process, favorable photostability, and low biotoxicity compared to conventional phosphorescent materials in recent years.^[^ ^1^ ^]^ The unique properties of carbon dot‐based RTP materials have made them highly versatile and sought‐after in various applications, including anti‐counterfeiting, information encryption, sensors, and bioimaging.^[^ ^2^ ^]^ These applications require a wide spectral range of phosphorescence emission, long‐lasting phosphorescence lifetime, and high quantum yield.^[^ ^3^ ^]^ In general, the achievement of RTP emission in conventional carbon dots remains challenging because of large nonradiative losses and the difficulties of its own excitons' intersystem crossing (ISC) between singly and triplet excited states.^[^ ^2^, ^4^ ^]^ Previous research has demonstrated that the introduction of heteroatoms, such as N,^[^ ^5^ ^]^ P,^[^ ^6^ ^]^ F,^[^ ^7^ ^]^ S,^[^ ^8^ ^]^ and others, can significantly enhance the ISC process in carbon dots, this enables the realization of RTP emission in carbon dots. The incorporation of heteroatoms has been seen to enhance the luminous centers and ISC processes inside carbon dots.^[^ ^4^, ^6^ ^]^ However, the phosphorescent emission of carbon dots is often characterized by a brief duration, mostly attributed to the quenching of triplet excited states resulting from non‐radiative loss pathways. In order to avoid the RTP extinguishment of carbon dots caused by the non‐radiative loss, attempts have been made to introduce polymers as the raw material for the synthesis of carbon dots, which can synthesize carbon dots with polymer‐like structural properties.^[^ ^9^ ^]^ The luminous groups in this distinctive structure are efficiently immobilized, resulting in the inhibition of nonradiative loss and enabling the realization of the RTP emission of carbon dots, thanks to the cross‐linking‐enhanced emission (CEE) effect.^[^ ^9^, ^10^ ^]^ Some researchers named these polymer‐structured carbon dots as carbonized polymer dots (CPDs).^[^ ^11^ ^]^ This approach opens up a variety of design options for the phosphorescent properties of CPDs, but these CPDs materials frequently do not have excellent afterglow times and lifetimes due to the limiting effect provided by the polymer structure is not sufficiently strong. In addition to the mentioned strategy, an alternative frequently utilized method to enhance the phosphorescent characteristics of carbon dots is through their incorporation within crystalline frameworks such as SiO_2_,^[^ ^12^ ^]^ cyanuric acid,^[^ ^8^, ^13^ ^]^ urea,^[^ ^14^ ^]^ boric acid/boron oxide,^[^ ^14^, ^15^ ^]^ zeolite,^[^ ^16^ ^]^ and inorganic metal salts,^[^ ^17^ ^]^ among others.^[^ ^18^ ^]^ Due to the rigid immobilized substrate that can effectively limit the nonradiative loss of carbon dots, this type of strategy can significantly enhance the RTP lifetime of carbon dots materials.^[^ ^19^ ^]^ However, this method makes it difficult to modulate the phosphorescent properties of the material, which tends to have only a single phosphorescent color emission, limiting the range of applications. The synthesis of carbon dots‐based phosphorescent materials with extended lifetimes and multiple emission colors through a single approach has proven to be a significant challenge.

In this study, we first present a novel strategy for achieving multi‐color ultralong room‐temperature phosphorescence. Our approach involves using precursors capable of generating multiple luminous centers, which then react with long‐chain polymers to produce carbonized polymer dots (CPDs) with immobilized luminescent centers. These CPDs are subsequently embedded into rigid matrices. The integration of long‐chain polymers during the synthesis process efficiently immobilizes the luminescent centers within the CPDs through crosslinking.^[^ ^4a^ ^]^ This immobilization, combined with the surrounding rigid matrix, synergistically limits the nonradiative loss of triple‐state excitons. As a result, the CPDs exhibit enhanced phosphorescent properties, including multicolor emission and a significantly prolonged phosphorescence lifetime at room temperature.

**Scheme 1:**
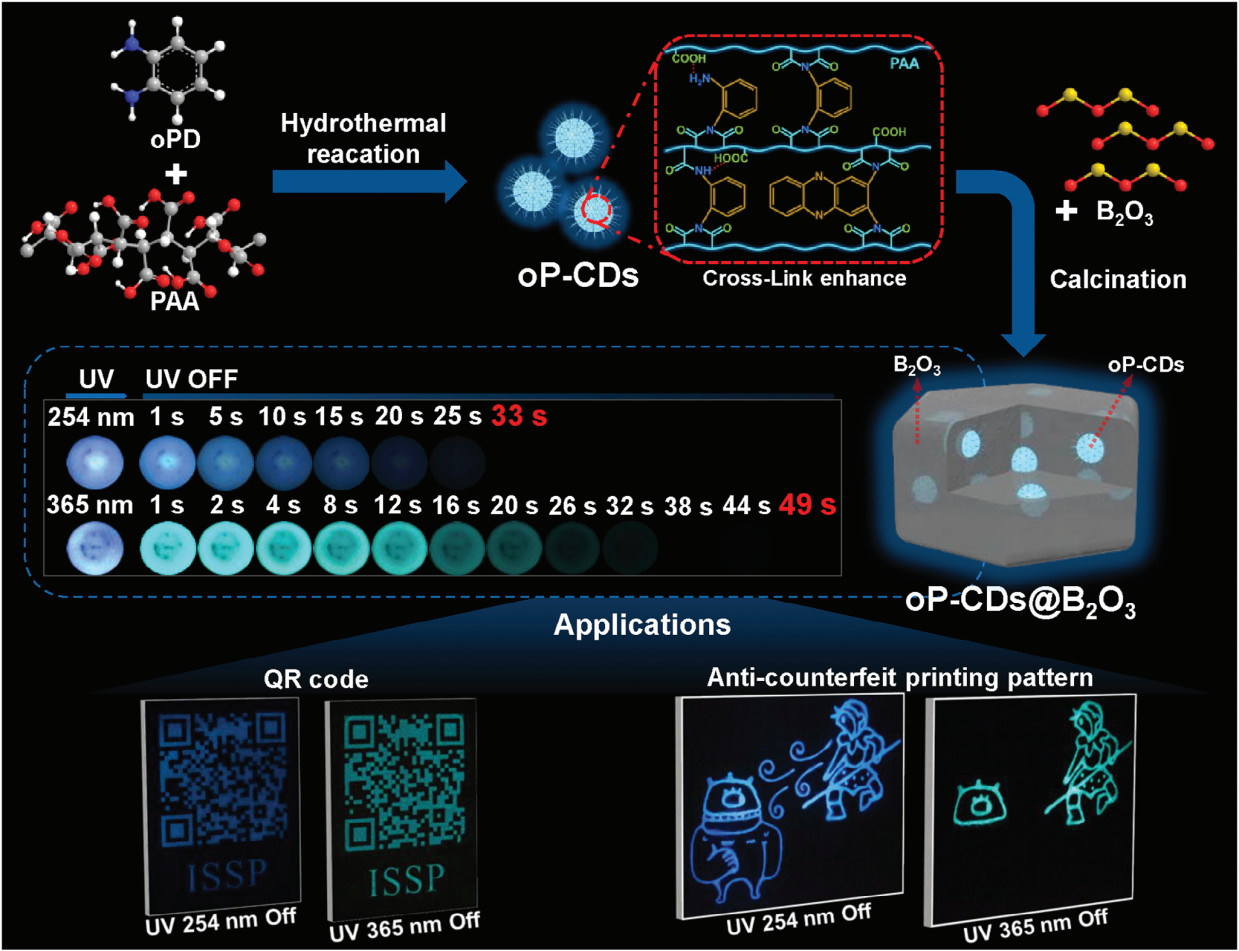
Schematic for the preparation and application of dual‐color phosphorescence oP‐CDs@B_2_O_3_ RTP materials.

For example, we prepared CPDs (named oP‐CDs) synthesized from o‐phenylenediamine and polyacrylic acid, which were embedded in a B_2_O_3_ matrix (**Scheme** **1**). The obtained CPDs‐based room‐temperature phosphorescent materials (named oP‐CDs@B_2_O_3_) demonstrate dual‐color phosphorescence emission, ultra‐long lifetime, and high phosphorescence quantum yields. The phosphorescence mechanism of this material has also been investigated. The use of the o‐phenylenediamine precursor introduces multiple luminous centers for the construction of oP‐CDs, as a consequence, the ensuing oP‐CDs@B_2_O_3_ exhibit phosphorescence emission in dual colors, ranging from blue to green. The polyacrylic acid precursor yields a cross‐linking‐enhanced emission (CEE) effect which can limit the nonradiative loss of oP‐CDs. The B_2_O_3_ matrix imposes constraints on the vibrational motion of oP‐CDs, by forming covalent bonds with the oP‐CDs and facilitating hydrogen bonding forces. These two effects synergistically diminish the nonradiative decay of the triplet state, leading to extended phosphorescence lifetimes and remarkably prolonged afterglow durations to the naked eye. Additionally, the material exhibits exceptional quantum yields. The phosphorescence lifetime of oP‐CDs@B_2_O_3_ was measured to be 3.62 s under 365 nm excitation. Furthermore, the oP‐CDs@B_2_O_3_ exhibits naked eye‐visible RTP glow time up to 49 s under 365 nm UV excitation, with a phosphorescence lifetime of 3.06 s for blue emission, and an RTP glow time visible to the naked eye for 33 s under 254 nm UV excitation. Moreover, this material displays a remarkable phosphorescence quantum yield of 19.5% when excited at 290 nm. The oP‐CDs@B_2_O_3_ material prepared has dual‐color phosphorescence emission, extremely long phosphorescence lifetime, and excellent phosphorescence quantum yield, which can satisfy the specifications for materials used in anti‐counterfeiting or information encryption. We also prepared and demonstrated anti‐counterfeiting and message encryption patterns using oP‐CDs@B_2_O_3_ material to verify this. The results indicate that this CPD‐based RTP material possesses significant potential for anti‐counterfeiting and information encryption applications.

## 2 Results and Discussion

**Figure 1:**
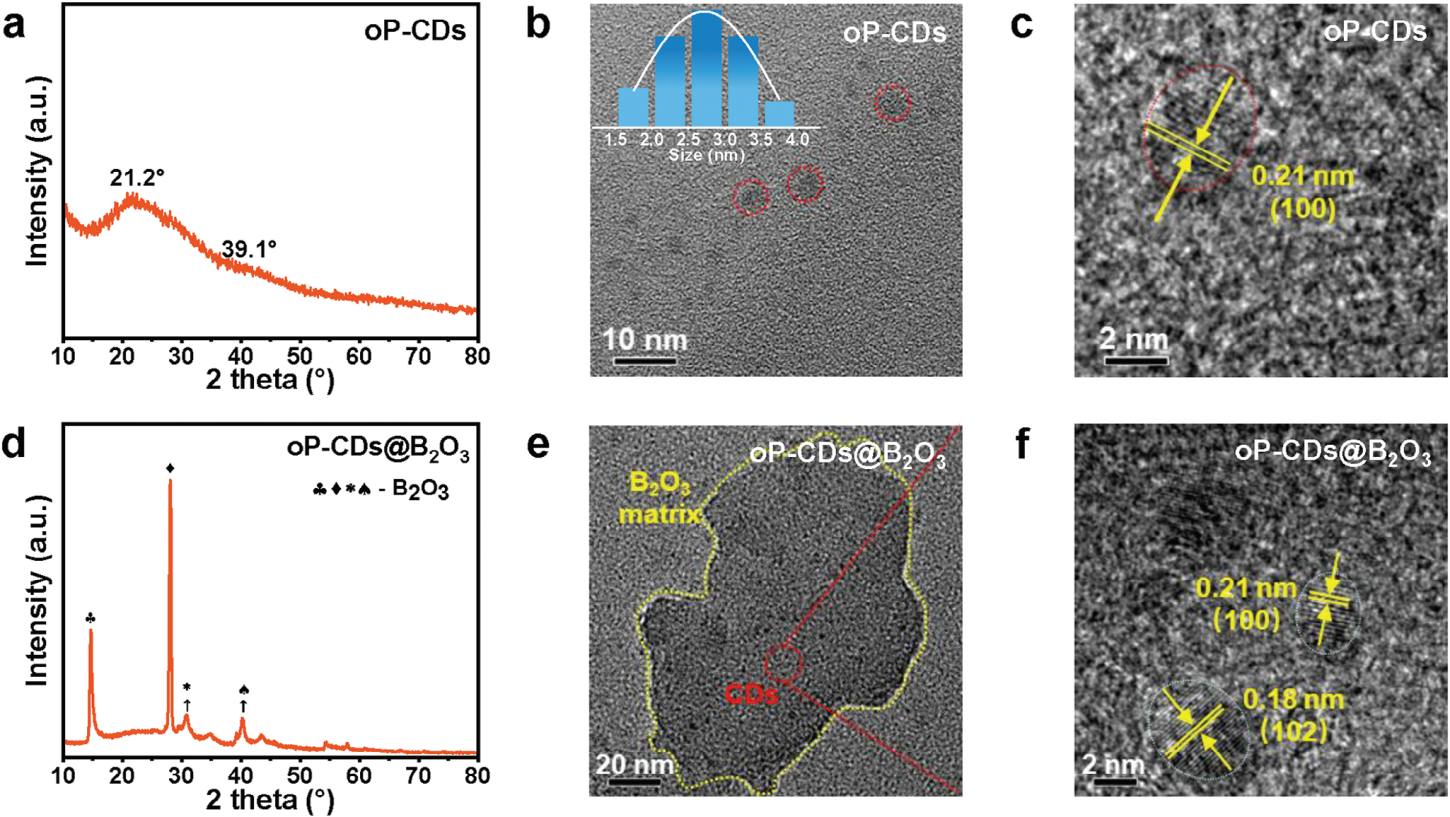
XRD and TEM characterization of oP‐CDs and oP‐CDs@B_2_O_3_. a) XRD pattern of oP‐CDs. b) TEM and c) HRTEM image of oP‐CDs (Inset: the size distribution of oP‐CDs). d) XRD pattern of oP‐CDs@B_2_O_3_. e) TEM and f) HR‐TEM image of oP‐CDs@B_2_O_3_.

The carbonized polymer dots (named oP‐CDs) were synthesized by a one‐step hydrothermal reaction approach employing o‐phenylenediamine and polyacrylic acid as precursor materials. The morphology and structure of the oP‐CDs were analyzed using TEM in **Figure** **1**b The TEM images revealed that the oP‐CDs exhibited a well‐dispersed quantum dot morphology, with an average diameter of 2.8 nm. Additionally, the HR‐TEM (Figure 1c) provided evidence of partially visible lattice stripes within the oP‐CDs, with a lattice spacing of 0.208 nm,^[^ ^20^ ^]^ this observation suggests the presence of graphitic. The XRD patterns (Figure 1a) show gentle bulging peaks in the graphite, which suggests that the carbon dots are not graphitized to a high degree overall, and it is assumed that the polyacrylic acid in the raw material has led to the structure of the oP‐CDs being more similar to that of the CPDs.^[^ ^9^ ^]^ Afterward, the oP‐CDs and B_2_O_3_ were thoroughly combined and subjected to heat treatment, resulting in the production of oP‐CDs@B_2_O_3_. The XRD pattern of oP‐CDs@B_2_O_3_ (Figure 1d) exhibited distinct peaks corresponding to the distinctive features of boron oxides suggesting the presence of B_2_O_3_, as well as the smooth and slightly elevated peaks associated with the oP‐CDs.^[^ ^12^, ^21^ ^]^ The dispersion of the oP‐CDs within the B_2_O_3_ matrix was evidenced by the TEM images presented in Figure 1e Furthermore, the assertion was corroborated by the use of HR‐TEM images, wherein the distinct lattice stripes of oP‐CDs dispersed inside the B_2_O_3_ matrix were readily discernible (Figure 1f).

**Figure 2:**
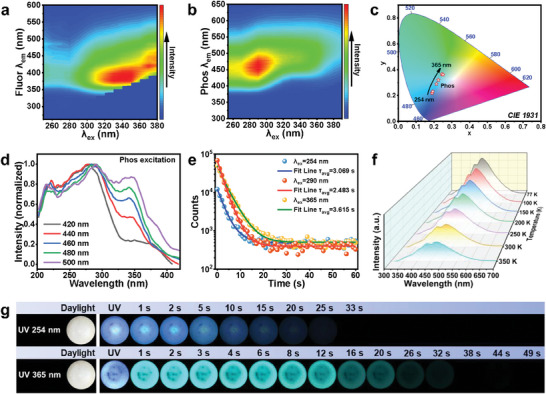
Photoluminescence characterization of oP‐CDs@B_2_O_3_. a) Fluorescent and b) Phosphorescent two‐dimensional excitation‐emission plot of oP‐CDs@B_2_O_3_. The luminous intensity rises with the color changing from blue to green and to red. c) CIE coordinates of the phosphorescence emission of oP‐CDs@B_2_O_3_ under different UV wavelengths from 254 to 365 nm. d) Photoluminescence excitation spectra of oP‐CDs@B_2_O_3_ with different phosphorescence emission. e) Time‐resolved phosphorescence decay and fitting curve of the emission bands at different wavelength excitation. f) Temperature‐dependent time‐resolved phosphorescence curves of oP‐CDs@B_2_O_3_. g) Photographs of oP‐CDs@B_2_O_3_ powders under daylight, excited with 254 and 365 nm UV lamp, and after removing UV.

Subsequently, the optical properties of oP‐CDs@B_2_O_3_ will be subjected to characterization. The phosphorescence spectrogram reveals that oP‐CDs@B_2_O_3_ exhibits fluorescence and phosphorescence within the excitation UV range of 254 nm‐380 nm (**Figure** **2**a,b). Notably, as the excitation wavelength increases, the phosphorescence transitions from a blue hue to a lime‐green hue. Figure 2c displays the CIE1931 color coordinates of the emission of phosphorescence. The phosphorescence excitation curves of the oP‐CDs@B_2_O_3_ (Figure 2d) indicate the presence of at least two distinct luminescent centers, each associated with the emission of blue and green phosphorescence, respectively. The phosphorescence decay spectra of the oP‐CDs@B_2_O_3_ were further measured as Figure 2e, and the decay spectral data were fitted to a two‐exponential function and calculated according to the following equation:

(1) $$\tau_{\mathrm{avg}}=\frac{\sum \alpha_{i}\tau_{i}^{2}}{\sum \alpha_{i}\tau_{i}}\;$$

The phosphorescence lifetime was determined to be 3.069, 2.483, and 3.615 s under excitation wavelengths of 254, 290, and 365 nm, respectively. Simultaneously, the quantum yields of phosphorescence were measured when subjected to stimulation by the aforementioned UV wavelengths. The corresponding values are presented in Table S1 (Supporting Information). The phosphorescence exhibited a maximum quantum yield of 19.5% when excited at a wavelength of 290 nm. Figure 2f illustrates the temperature‐dependent phosphorescence spectra of oP‐CDs@B_2_O_3_. The phosphorescent emission of oP‐CDs under 290 nm excitation gradually decreases as the temperature increases from 77 to 350 K. This behavior is characteristic of phosphorescent materials, where lower temperatures restrict molecular vibrations, resulting in reduced non‐radiative losses and a stronger afterglow emission.^[^ ^21^ ^]^ Notably, oP‐CDs@B_2_O_3_ exhibits an exceptional afterglow lifetime visible to the naked eye, as shown in Figure 2g and Videos S1 and S2 (Supporting Information). Under 254 nm UV excitation, the blue afterglow remains visible for an impressive 33 s, while under 365 nm UV excitation, the lime green afterglow persists for an extraordinary 49 s. This extended afterglow duration is noteworthy, as it represents one of the lengthiest known periods of afterglow among CD‐based RTP materials (Table S2, Supporting Information).

**Figure 3:**
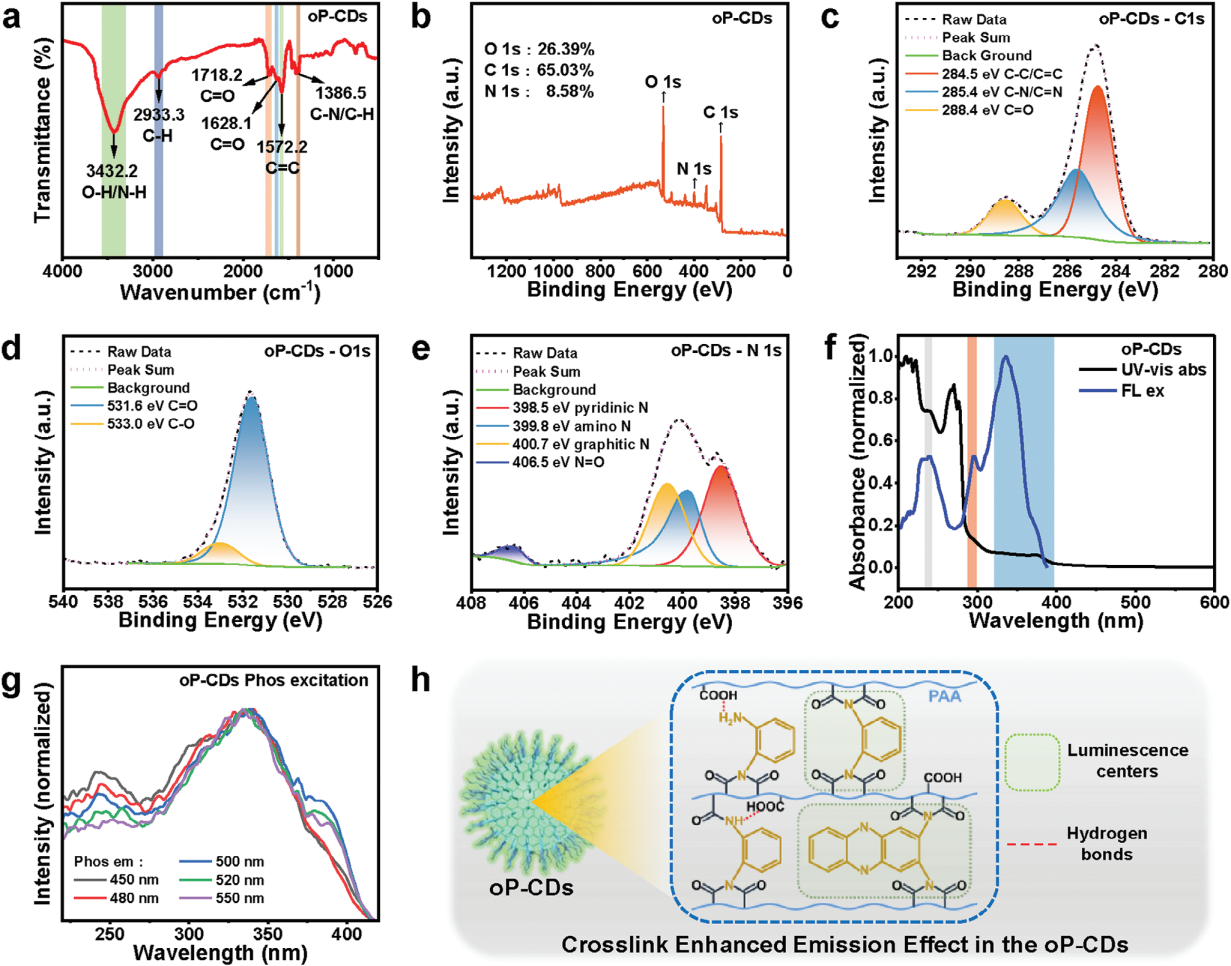
Characterization of oP‐CDs. a) FT‐IR spectra of oP‐CDs. b) XPS spectra and c) C 1s HR‐XPS spectra, d) O 1s HR‐XPS spectra, e) N 1s HR‐XPS spectra of oP‐CDs. f) UV–vis absorption spectra and fluorescence excitation of oP‐CDs. g) Photoluminescence excitation spectra of oP‐CDs with different phosphorescence emission. h) The cross‐linking‐enhanced emission (CEE) effect of oP‐CDs.

To investigate the impact of o‐phenylenediamine on the structure and optical properties, we conducted a comprehensive analysis of the chemical bonding (**Figure** **3**) and optical properties of the oP‐CDs (Figure S1, Supporting Information). The FT‐IR spectral curves (Figure 3a) show that the C═O stretching vibration of the carbonyl group in carboxylic acids is represented by the peak at 1718.2 cm^−1^, the stretching and bending vibration of C─H is related to the absorption peak at 2933 cm^−1^, the carbonyl group stretching vibration of amide (amide I‐band), C═O and the C═N stretching vibration are related to the absorption peak at 1628.1 cm^−1^, and the C═C stretching vibration is represented by the peak at 1572 cm^−1^. These data show that amide bonds are formed between o‐phenylenediamine and polyacrylic acid during the hydrothermal reaction.^[^ ^9^, ^22^ ^]^ By using XPS to characterize the bonding information of the oP‐CDs, as illustrated in Figure 3b, this inference was further confirmed. The N element is also present in the carbon dots, as seen by the distinctive peaks at 285.0, 400.1, and 530.6 eV, which correspond to C 1s, N 1s, and O 1s, respectively. The computed concentrations of these three elements are 65.03%, 8.58%, and 26.39%, respectively. Upon analyzing the elemental peaks, it becomes evident that the C 1s spectrum (Figure 3c) corresponds to C─C/C═C bonds at 284.5 eV, C─N/C═N bonds at 285.4 eV, and C═O bonds at 288.4 eV. The O 1s peaks (Figure 3d) have binding energies of 531.6 eV corresponding to C═O bonds and 533.0 eV corresponding to C─O bonds. The N1s peaks (Figure 3e) exhibit distinct binding energies, namely 398.5 eV for pyridinic N, 399.8 eV for amino N, 400.7 eV for graphitic N, and a little contribution at 406.5 eV attributed to N═O bonds.^[^ ^9^ ^]^ XPS and FT‐IR spectra together demonstrate the formation of amide bonds and oxidation groups inside the oP‐CDs due to the incorporation of o‐phenylenediamine. These structures and groups were recognized as significant luminous centers for RTP emission of CDs‐based materials and the essential part of the cross‐linking‐enhanced emission effect ^[^ ^23^ ^]^ (Figure 3h).

To analyze the luminous centers present in oP‐CDs, the UV–vis absorption spectra were examined, as depicted in Figure 3f. The spectra reveal distinctive absorption peaks at 270 nm and broad absorption peaks at 376 nm, corresponding to the π→π* transitions of the C═C bond and the n→π* transitions of the C═O/C═N bond, respectively.^[^ ^23^, ^24^ ^]^ It is commonly believed that the first absorption peak arises from the internal carbon core of the carbon dots, while the second peak is associated with the doping of nitrogen into the interior of the carbon dots, forming either a graphite N or pyridine N structure. By studying the phosphorescence excitation spectra (Figure 3g), it is observed that oP‐CDs exhibit two distinct excitation peaks at 240 nm and 350 nm. This suggests the existence of two separate phosphorescence centers within the oP‐CDs. Based on the UV–vis absorption spectra, it is speculated that these two excitation centers are associated with the C═C bond and the C═O/C═N bond, respectively. Additionally, it is notable that as the wavelength of the excitation light source increases, the phosphorescent color tends to shift towards green. This observation suggests that the excitation peak around 350 nm corresponds to the luminescent center responsible for green phosphorescence. Conversely, the luminescent center near 240 nm is associated with blue phosphorescence. These findings indicate that the inclusion of o‐phenylenediamine introduces multiple luminescent centers to the oP‐CDs, resulting in dual‐color phosphorescence emission.^[^ ^24^ ^]^

**Figure 4:**
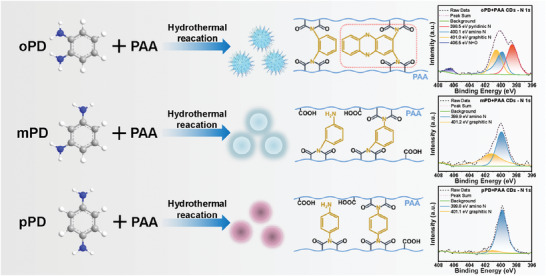
Effects of different positions of the amino group on the benzene ring on the structure and phosphorescence properties of CDs.

In order to further validate the role of o‐phenylenediamine, m‐phenylenediamine and p‐phenylenediamine were substituted in the synthesis of carbon dots, resulting in the formation of mP‐CDs and pP‐CDs, respectively. It was observed that mP‐CDs did not exhibit any phosphorescence, while pP‐CDs displayed a weak and short‐lived phosphorescence visible to the naked eye (Figure S2, Supporting Information). XPS spectra analysis revealed the presence of carbon, oxygen, and nitrogen elements in all three sample groups (Figures S3 and S4, Supporting Information). Notably, the mP‐CDs and pP‐CDs samples exhibited a lower nitrogen content (5.04% and 5.74% respectively) compared to the oP‐CDs sample group. It is widely acknowledged that a higher concentration of elemental nitrogen positively influences the phosphorescent properties of carbon dots. Further examination of chemical bonding through peak splitting in the C 1s and N 1s characteristic peaks showed that oP‐CDs prominently featured pyridinic N, distinguishing them from the other two categories of CDs. The positioning of the amino group on the benzene ring played a crucial role in the generation of pyridinic N structure in oP‐CDs, making o‐phenylenediamine more suitable for N doping in CDs compared to m‐phenylenediamine and p‐phenylenediamine (**Figure** **4**).^[^ ^25^ ^]^ This is the primary reason for the strongest phosphorescent characteristics observed in oP‐CDs. The presence of two adjacent amino groups on the benzene ring facilitated the introduction of N into the interior of CDs in the form of pyridinium N.^[^ ^26^ ^]^ This process promoted n→π* transitions, enhancing the transition from singlet to triplet states and facilitating room temperature phosphorescence generation.^[^ ^10^, ^27^ ^]^

Based on characterization data, we contend that the incorporation of polymer PAA results in the formation of an amide and hydrogen bonding structure with o‐phenylenediamine. This interaction allows the PAA polymer to function akin to a skeletal framework, thereby effectively stabilizing the excited triplet state and mitigating non‐radiative losses through the cross‐linking‐enhanced emission (CEE) effect.^[^ ^9^, ^10^, ^12^ ^]^ With the objective of validating the role of the long‐chain structure of polyacrylic acid, different CDs/CPDs were synthesized as reference groups by using oPD&AA (named oA‐CDs), oPD (named o‐CDs), or PAA (named P‐CDs). oA‐CDs demonstrate a mild RTP emission due to the absence of long‐chain polymer PAA as a raw material to induce the CEE effect (Figure S6, Supporting Information). It is worth noting that both o‐CDs and P‐CDs consist of a single raw material that lacks the ability to form an amide bond during the reaction. Consequently, o‐CDs and P‐CDs exhibit no phosphorescence or only weak phosphorescence intensity, respectively (Figure S6, Supporting Information). This observation is further supported by the bonding information obtained from FT‐IR and XPS analysis (Figures S7–S10, Supporting Information).

**Figure 5:**
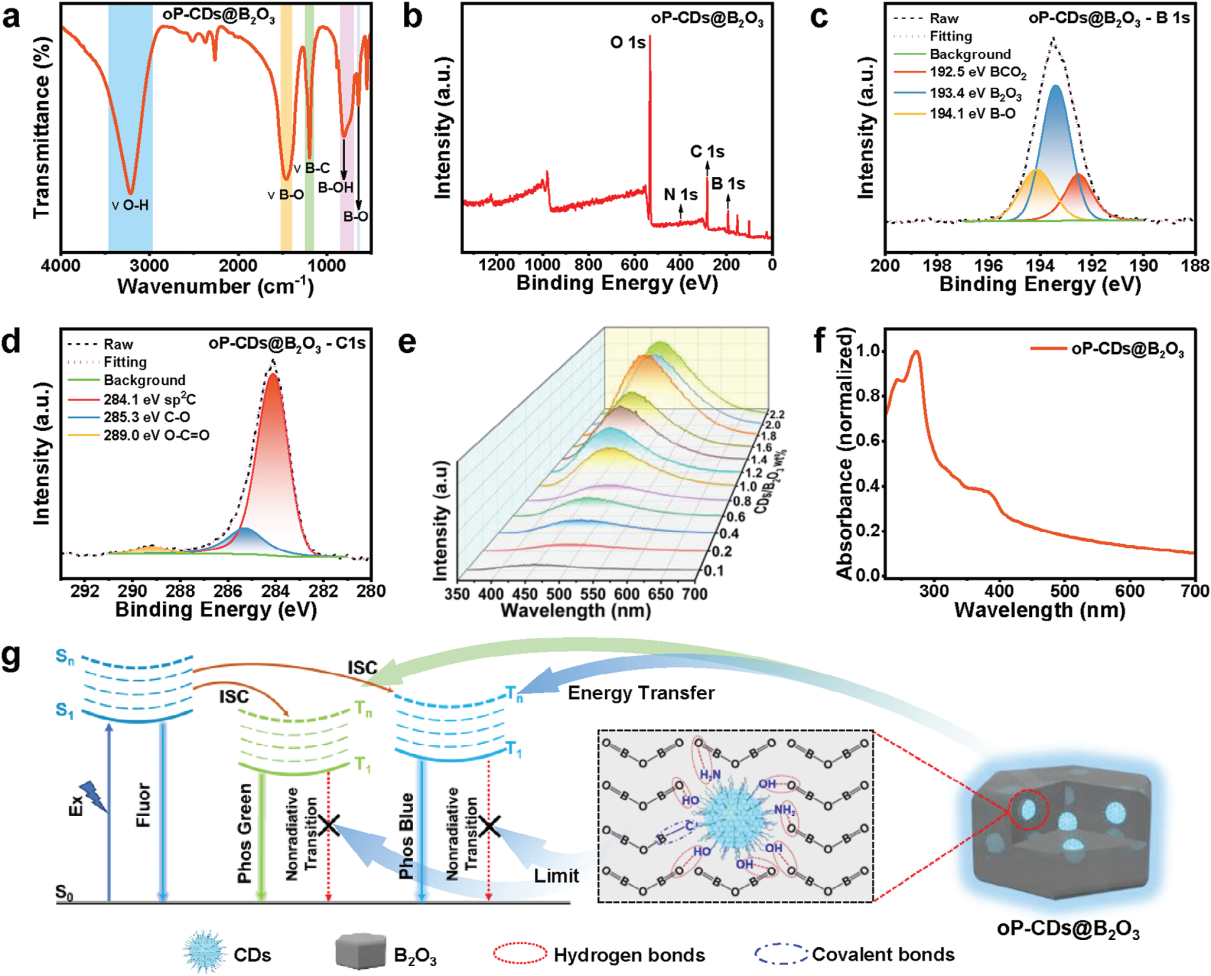
Characterization of oP‐CDs@B_2_O_3_. a) FT‐IR spectra of oP‐CDs@B_2_O_3_. b) XPS spectra, c) B 1s HR‐XPS spectra and d) C 1s HR‐XPS spectra of oP‐CDs@B_2_O_3_. e) Phosphorescence curves of oP‐CDs@B_2_O_3_ with different oP‐CDs contents under 290 nm excitation. f) UV–vis absorption spectra and fluorescence excitation of oP‐CDs@B_2_O_3_. g) Schematic structure of oP‐CDs@B_2_O_3_ and effect on phosphorescence properties.

To understand the functions of B_2_O_3_ matrices, we have conducted further characterization of the oP‐CDs@B_2_O_3_. According to the analysis of the FT‐IR spectra in **Figure** **5**a, the absorption peaks located at 3215 and 805 cm^−1^ can be attributed to the stretching vibrations of the O─H and B─OH bonds, respectively. Similarly, the peaks observed at 1451 and 640 cm^−1^ correspond to the stretching vibrations of the B─O bond. Furthermore, the absorption peak detected at 1195 cm^−1^ is indicative of the presence of the B─C bond.^[^ ^28^ ^]^ The XPS data (presented in Figure 5b–d) illustrates the observed peak‐splitting phenomena for elements B and C. The B 1s spectrum exhibits distinct peaks at 192.5 eV for BCO_2_, 193.4 eV for B_2_O_3_, and 194.1 eV for B─O bonds.^[^ ^14^, ^15^ ^]^ Likewise, the C 1s spectrum displays peaks at 284.1 eV for sp2 C, 285.3 eV for C─O bonds, and 289.0 eV for O─C═O bonds. It is observed that the intensity of the fitted peaks associated with B─C in the B 1s spectrum of XPS increases compared to the fractionation peaks of B_2_O_3_.^[^ ^14^, ^29^ ^]^ The XPS data, in conjunction with the FT‐IR data, indicates the formation of covalent bonds between the electron‐absorbing boron atoms and the oP‐CDs. These covalent bonds allow the B_2_O_3_ matrix to immobilize the oP‐CDs effectively, resulting in a notable reduction in the nonradiative loss of triplet excitons ^[^ ^30^ ^]^ (Figure 5g). Consequently, this immobilization strategy enables the enhancement of RTP emission. It is worth mentioning that B_2_O_3_ exhibits a certain level of phosphorescence at room temperature (Figure S11, Supporting Information). The phosphorescence emission from the embedded CPDs is then transferred to the boron oxide shell through phosphorescence energy transfer. This transfer mechanism helps reduce the phosphorescence energy loss caused by the rigid matrix encapsulation, thereby further enhancing the phosphorescence emission of oP‐CDs@B_2_O_3_.^[^ ^14^ ^]^

Furthermore, we conducted experiments using samples containing varying concentrations of oP‐CDs@B_2_O_3_ as Figure 5e. Observations revealed a gradual increase in the intensity of phosphorescence emission as the concentration of oP‐CDs increased. However, once a certain proportion of oP‐CDs was reached, the phosphorescence intensity remained relatively constant. This phenomenon can be attributed to the limited capacity of the matrix to effectively immobilized oP‐CDs. Notably, comparing the phosphorescence emission curves of oP‐CDs and oP‐CDs@B_2_O_3_, suggests that the incorporation of oP‐CDs into B_2_O_3_ did not alter the phosphorescent characteristics of the material other than contributing to a blue shift in phosphorescence (Figure S1f, Supporting Information).

As a comparison, we embedded oA‐CDs (synthesized from o‐phenylenediamine and acrylic acid) without polymer long chains in a B_2_O_3_ matrix, which has very weak phosphorescence emission (less than 7 s, as shown in Figure S12, Supporting Information). Based on this, we propose that the synergy of the rigid B_2_O_3_ matrix and the polymer cross‐linking effect in the oP‐CDs serves to restrict the dissipation of excitons in the triplet state, thereby enabling the attainment of this oP‐CDs@B_2_O_3_ material with an extended afterglow duration.

**Figure 6:**
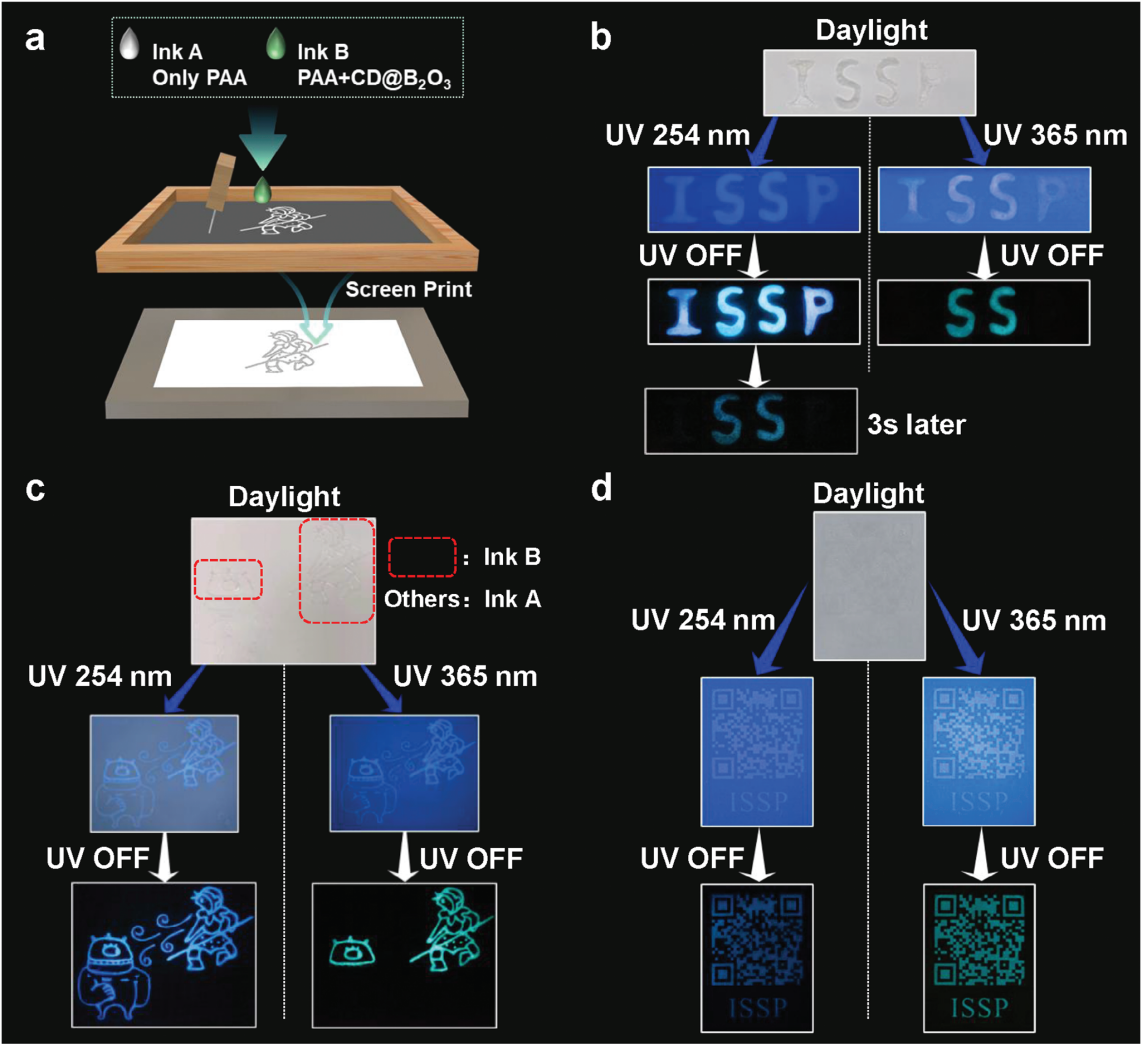
Demonstration of oP‐CDs@B_2_O_3_ for apply in the field of information encryption and anti‐counterfeiting. a) Schematic of screen printing using Ink A and Ink B. Digital photograph of b) ISSP pattern made by Ink A & Ink B under daylight, 254 and 365 nm UV light and after removing UV. c) Anti‐counterfeit printing pattern made by Ink A & Ink B under daylight, 254 nm & 365 nm UV light and after removing UV. d) QR code made by Ink B under daylight, 254 nm & 365 nm UV light and after removing UV.

Owing to the unusual ultralong dual‐emission phosphorescence features of oP‐CDs@B_2_O_3_ materials, it has attractive prospects in fields such as anti‐counterfeiting and information encryption. These applications can be easily realized through simple screen printing techniques (**Figure** **6**a). Ink A, composed of a PAA solution, and Ink B, a mixture of PAA solution and oP‐CDs@B_2_O_3_ powder, were used for printing. Ink A produced a printed pattern with discernible blue fluorescence and a few seconds‐long blue phosphorescence after drying. The patterns shown in Figure 6b to d were all produced through screen printing. The letters ISSP were printed using both Ink A and Ink B, with Ink A printing the letters I and P, while Ink B printed the central letters SS. Under 254 nm excitation, the blue fluorescent letters “ISSP” were observed followed by a blue phosphorescent pattern after removing the UV light source. Similarly, under 365 nm excitation, the blue fluorescent letters “ISSP” were observed, with only the letters “SS” showing a green phosphorescent pattern after removing the UV light. Using the same technique, the Monkey King and the one‐eyed monster were replicated using Ink A and Ink B (Figure 6c). Under both 254 and 365 nm UV excitation, a blue fluorescent pattern was observed, followed by a blue phosphorescent pattern after removing the 254 nm UV light. Under 365 nm UV excitation and subsequent removal of the light source, a green phosphorescent pattern appeared. Anticounterfeiting QR codes were also produced using screen printing (Figure 6d). After 254 nm UV light exposure, the QR code patterns displayed blue fluorescence. Following the cessation of excitation at 254 nm, blue phosphorescent QR code patterns became visible. Additionally, under 365 nm UV light, blue fluorescence was observed, which transitioned into a green phosphorescent pattern after removing the 365 nm UV light. Importantly, even after being stored at room temperature (20 °C) for a duration of six months, the phosphorescent properties of the QR code pattern remained unaffected. This result highlights the remarkable light stability of our inks (Figure S14, Supporting Information).

## 3 Conclusion

In summary, a novel room‐temperature phosphorescent CDs‐based material with dual emission in blue and lime green wavelengths, long lifetime, exceptional quantum yield, and very long afterglow time under naked eye observation has been prepared. Under room temperature, the oP‐CDs@B_2_O_3_ material exhibits a phosphorescent color that changes from blue to lime green with the redshift of the excitation wavelength. Multiple luminescent centers were introduced in CPDs through the incorporation of polymer PAA and o‐phenylenediamine with a benzene ring as precursor materials. The incorporation of polymer‐like structures into the carbon dots led to the activation of a cross‐linking‐enhanced emission effect. This effect, in combination with the constraint provided by the rigid B_2_O_3_ substrate, effectively minimized the nonradiative loss of the oP‐CDs. Consequently, the oP‐CDs@B_2_O_3_ material exhibited exceptional phosphorescence lifetimes and quantum yields. The phosphorescence lifetime under 254 nm excitation is 3.069 s, while under 365 nm excitation, it is 3.615 s. Remarkably, the naked eye can observe blue phosphorescence with an afterglow of up to 33 s under 254 nm excitation, and green phosphorescence for an astonishing 49 s under 365 nm excitation. Additionally, the phosphorescence quantum yield reaches a high efficiency, potentially up to 19.5% when excited at 290 nm. This study has showcased the practical applications of these remarkable phosphorescent properties in the domains of anticounterfeiting and information encryption. It is anticipated that this material will find diverse applications in anticounterfeiting measures, information encryption protocols, fingerprint identification systems, and display technologies.

## 4 Experimental Section

Essential Experimental Procedures/Data are available in the Supporting Information of this article.

## Conflict of Interest

The authors declare no conflict of interest.

## Supporting information

Supporting Information

Supplemental Video 1

Supplemental Video 2

## Data Availability

The data that support the findings of this study are available in the Supporting Information of this article.
