# Supplementary material for: Multiemitting Ultralong Phosphorescent Carbonized Polymer Dots via Synergistic Enhancement Structure Design
Source: Adv Sci (Weinh). 2024 Mar 29;11(18):2400781. doi: 10.1002/advs.202400781 (PMC11095232; doi:10.1002/advs.202400781)
Supplement: Supplementary file 1 — Supporting Information [file ADVS-11-2400781-s001.pdf]

ADVANCED SCIENCE
Open Access

Supporting Information

for *Adv. Sci.*, DOI 10.1002/advs.202400781

Multiemitting Ultralong Phosphorescent Carbonized Polymer Dots via Synergistic Enhancement Structure Design

Qipeng Zhang, Shihao Xu, Lanpeng Zhang, Liang Yang and Changlong Jiang**

Supporting Information

Multi-emitting ultralong phosphorescent carbonized polymer dots via synergistic enhancement structure design

Qipeng Zhang, Shihao Xu, Lanpeng Zhang, Liang Yang* and Changlong Jiang*

Abstract: Advancing a metal-free room temperature phosphorescent (RTP) material that exhibits multi-color emission, remarkable RTP lifetime, and high quantum yield still faces the challenge of achieving intersystem crossing between singly and triplet excited states, as well as the rapid decay of triplet excited states due to nonradiative losses. In this study, we propose a novel strategy to address these limitations by incorporating o-phenylenediamine, which generates multiple luminescent centers, and long-chain polyacrylic acid to synthesize carbonized polymer dots (CPDs). These CPDs are then embedded in a rigid B_2O_3 matrix, effectively limiting non-radiative losses through the synergistic effects of polymer cross-linking and the rigid matrix. The resulting CPDs-based materials exhibit remarkable ultralong phosphorescence in shades of blue and lime green, with a visible lifetime of up to 49 seconds and a high phosphorescence quantum yield. Simultaneously, this study demonstrates the practical applicability of these excellent material properties in anti-counterfeiting and information encryption.

Contents

1. Materials and Methods

2. Synthesis

3. Preparation of Ink for Printing

4. Supplementary Figures (Figure S1-S12)

5. Supplementary Tables (Table S1-S2)

6. References

7. Author Contributions

1. Materials and Methods

Materials: Polyacrylic acid (molecular weight: 3000, 50 wt%), o-phenylenediamine, m-phenylenediamine, p-phenylenediamine, acrylic acid, and boron oxide were purchased from aladdin chemicals. Deionized (DI) water used in the experiments was prepared by the laboratory deionized water machine. All raw materials were not purified unless otherwise mentioned.

Materials characterization: TEM and HRTEM were taken by JEM-2100F of Nippon Electronics Corporation.XRD characterization was obtained by Rigaku Smartlab 9kW. Nicolet iS10 FT-IR of Thermo Scientific measured FT-IR, XPS was measured by ESCALAB 250Xi of Thermo Scientific, and Shimadzu UV-1900i, measured the UV-vis solution portion and solid powders were measured by Shimadzu 3600-plus. ^{1}H NMR ^{13}C NMR and ^{19}F NMR were recorded on a Bruker-500MHz Spectrometer (^{1}H NMR: 500MHz, ^{13}C NMR: 125MHz, ^{19}F NMR: 470MHz,) using TMS as internal reference. The chemical shifts (δ) and coupling constants (*J*) were expressed in ppm and Hz. Phosphorescence and fluorescence spectra were measured by an Agilent G9800A fluorometer, and both phosphorescence lifetime curves and variable temperature phosphorescence spectra were measured by an Edinburgh FLS-1000 fluorometer. Phosphorescence quantum yields were measured by an Einburgh Instruments FS 5 fluorometer with an integrating sphere. Phosphorescence measurements were set to a delay time of 0.1 ms. Digital photos and demonstration videos were taken by smartphone and Nikon Z30.

2. Synthesis

Synthesis of oP-CDs: O-phenylenediamine (0.054 g) was dissolved in 20 mL of DI water and 0.216 g of polyacrylic acid was added, and the resulting solution was transferred to a Teflon-lined autoclave (50 mL) and heated for 600 mins at 200 °C. The solution in the autoclave was then removed and dialyzed in 5000 Da dialysis bags in deionized water for 24 h (with water changes every 8 h). The solution was then centrifuged in a high-speed centrifuge at 11000 rpm for 20 min to remove the precipitate, and the supernatant was filtered through a 0.22-micron membrane and freeze-dried to obtain the carbon dots powder sample. The final product obtained oP-CDs was passed by the Preparative Thin-Layer Chromatography (PrepTLC) method, try to isolate the fluorescent small molecules and was examined by NMR in order to exclude the effect of fluorescent molecules (Figure S13).

Synthesis of other CDs: The synthesis method was the same as that of oP-CDs, and the raw materials were replaced with (1) o-phenylenediamine 0.054 g and acrylic acid 0.216 g; (2) o-phenylenediamine 0.054 g; (3) polyacrylic acid 0.216 g, (4) m-phenylenediamine 0.054 g and polyacrylic acid 0.216 g, (5) p-phenylenediamine 0.054 g and polyacrylic acid 0.216 g, respectively.

Synthesis of oP-CDs@B_2O_3: Different masses of carbon dots were dissolved in 30 mL of deionized water with 1 g of B_2O_3 powder, respectively, and the beaker containing the solution was heated and stirred at 60 °C until the solution was completely evaporated, after which the powder was taken out and put into an onyx mortar and ground uniformly, and the powder was transferred to a ceramic crucible and heated at 200 °C for 180 min to obtain oP-CDs@B_2O_3.

3. Preparation of Ink for Printing

Ink A: directly use the purchased 50 wt% polyacrylic acid solution.
Ink B: The obtained oP-CDs@B_2O_3 was ground into powder using ball ink and passed through a 200-mesh stainless steel sieve, and the powder was mixed with the polyacrylic acid solution at a mass ratio of 1:2 to obtain Ink B.
Printing Size: All patterns are evenly brushed on standard A4 papers (21 cm×29.7 cm). Printed two-color phosphorescent letters "ISSP" measure 3.5 cm×12.5 cm, the size of the QR code is 6 cm×6 cm, and the Monkey King is printed in a rectangular frame measuring 18 cm high by 24 cm wide.
After the printing pattern is completed the paper and ink raw materials are stored in a room environment at a constant temperature of 20 °C, without direct sunlight.

4. Supplementary Figures

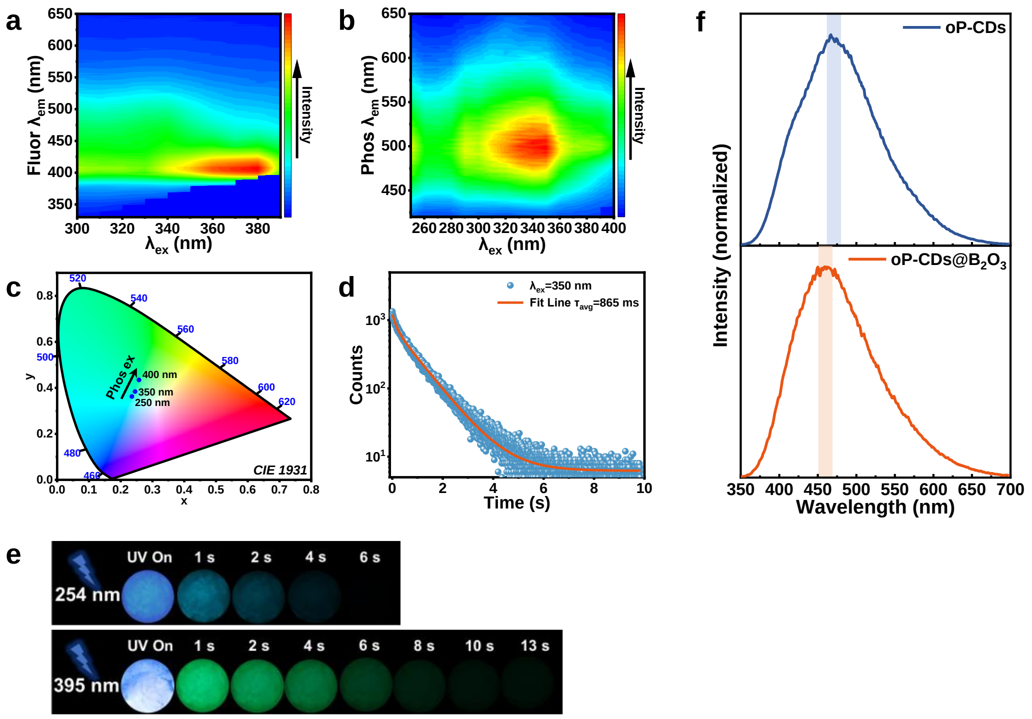

Figure S1. Photoluminescent properties of oP-CDs. a, Fluorescent and b, Phosphorescent two-dimensional excitation-emission plot of oP-CDs. The luminous intensity rises with the colour changing from blue to green and to red. c, CIE coordinates of the phosphorescence emission of oP-CDs under 254 nm, 350 nm and 400 nm excitation. d, Time-resolved phosphorescence decay and fitting curve of the emission bands at 500 nm with 350 nm excitation. e, Photographs of oP-CDs under daylight, excited with 254 nm & 365 nm UV lamp, and after removing UV. f, Comparison of phosphorescence emission of oP-CDs and oP-CDs@B_2O_3 under excitation of a 290 nm UV.

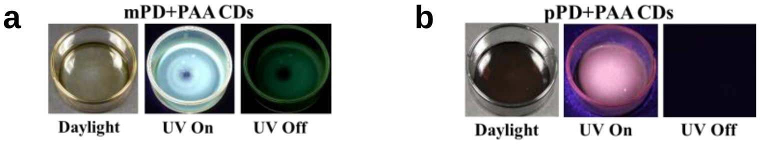

Figure S2. Photographs of mPD&PAA-CDs and pPD&PAA-CDs. a, mPD&PAA-CDs, b, pPD&PAA-CDs under daylight, excited with 365 nm UV lamp, and after removing UV.

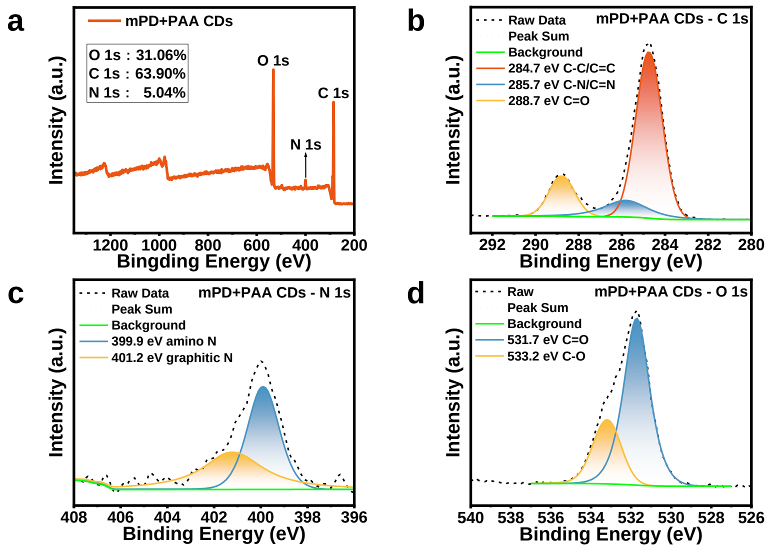

Figure S3. XPS characterization of mPD&PAA-CDs. a, XPS spectra. b, C 1s HR-XPS spectra. c, N 1s HR-XPS spectra. d, O 1s HR-XPS spectra.

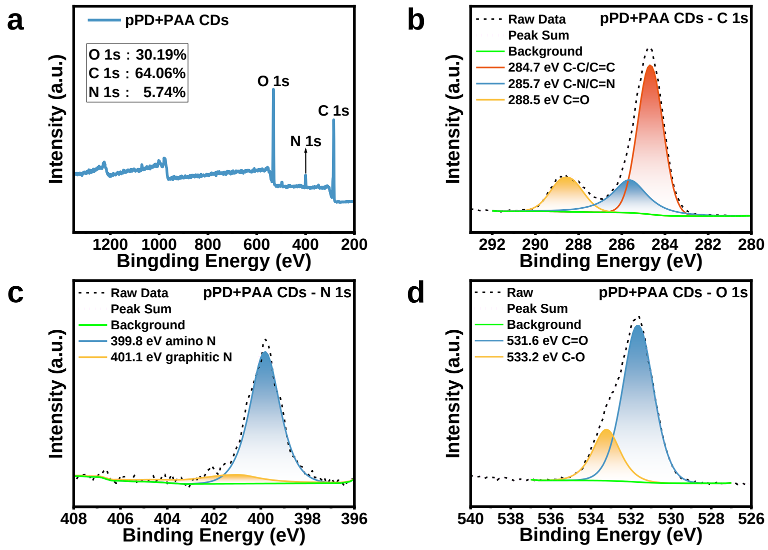

Figure S4. XPS characterization of pPD&PAA-CDs. a, XPS spectra. b, C 1s HR-XPS spectra. c, N 1s HR-XPS spectra. d, O 1s HR-XPS spectra.

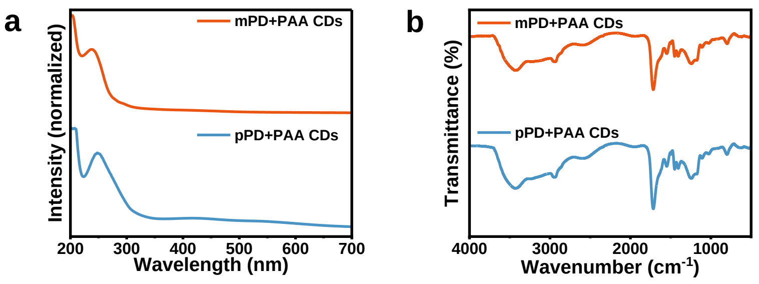

Figure S5. Characterization of mPD&PAA and pPD&PAA-CDs. a, UV-vis absorption spectra. b, FT-IR spectra.

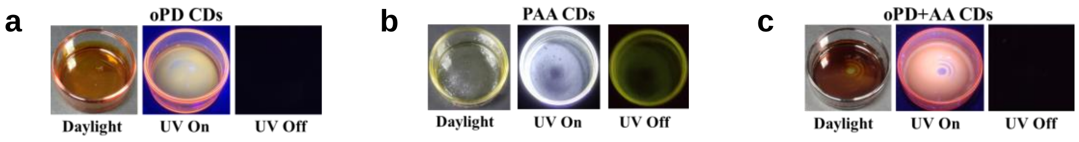

Figure S6. Photographs of o-CDs, p-CDs and oA-CDs. a, oPD&AA-CDs, b, PAA-CDs, c, oPD-CDs under daylight, excited with 365 nm UV lamp, and after removing UV.

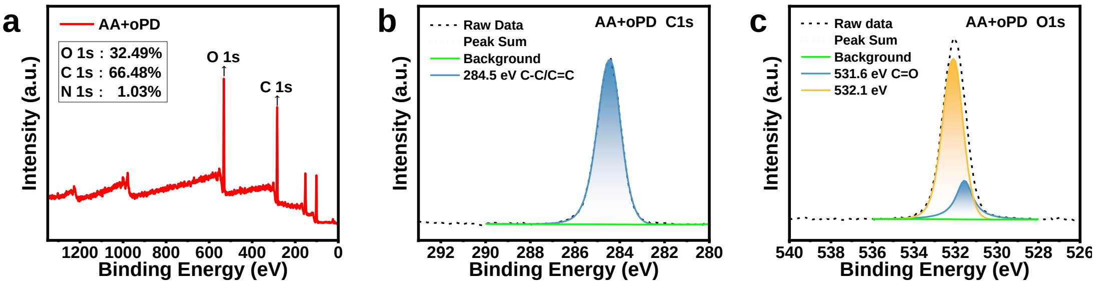

Figure S7. XPS Characterization of AA&oPD-CDs. a, XPS spectra. b, C 1s HR-XPS spectra. c, O 1s HR-XPS spectra.

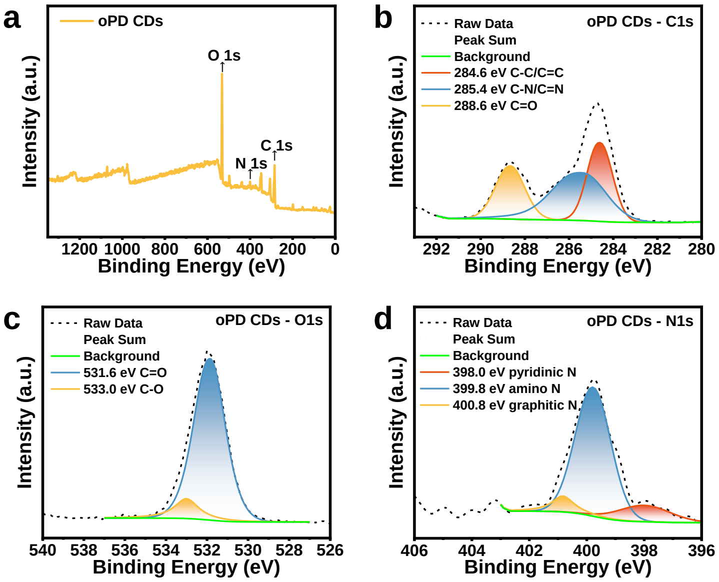

Figure S8. XPS Characterization of oPD-CDs. a, XPS spectra. b, C 1s HR-XPS spectra. c, N 1s HR-XPS spectra. d, O 1s HR-XPS spectra.

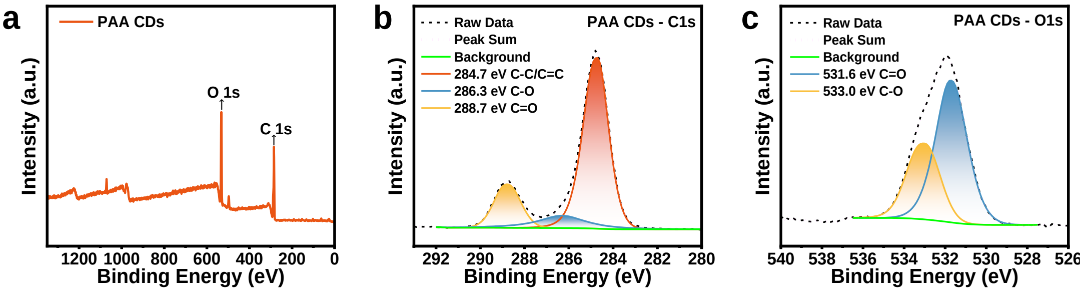

Figure S9. XPS Characterization of PAA-CDs. a, XPS spectra. b, C 1s HR-XPS spectra. c, O 1s HR-XPS spectra.

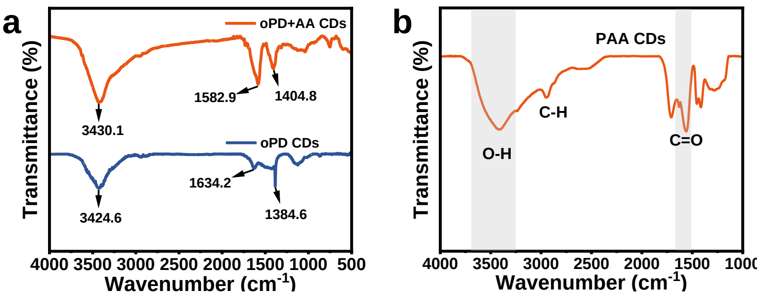

Figure S10. FT-IR Characterization of o-CDs, p-CDs and oA-CDs. a, FT-IR spectra of two CDs prepared from oPD&AA, oPD respectively. b, FT-IR spectra of PAA-CDs.

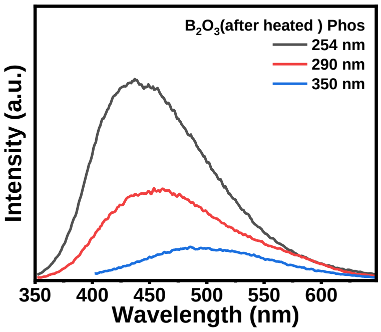

Figure S11. Phosphorescence emission of B_2O_3 after heat treatment under different UV excitations.

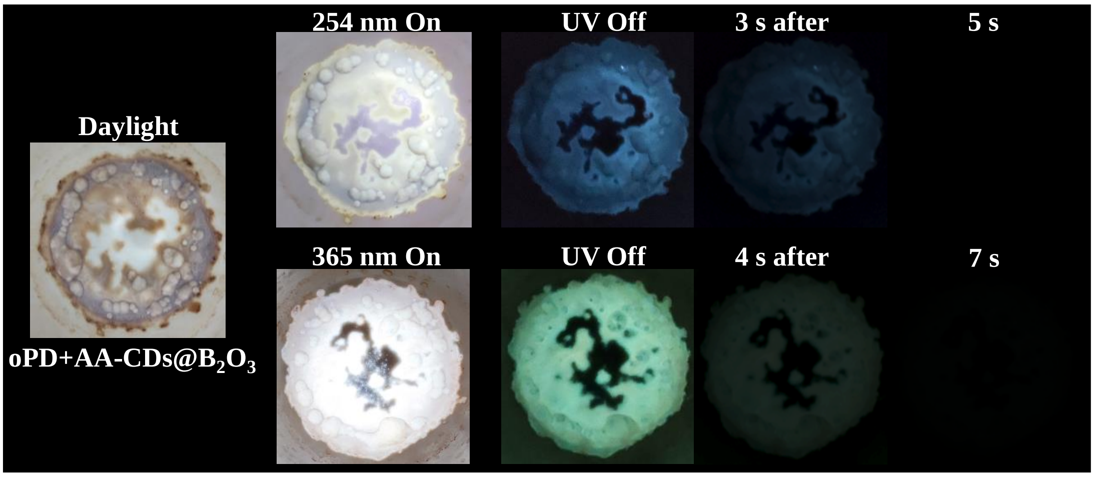

Figure S12. Photographs of oPD&AA-CDs@B_2O_3 under daylight, excited with 254 nm & 365 nm UV lamp, and after removing UV.

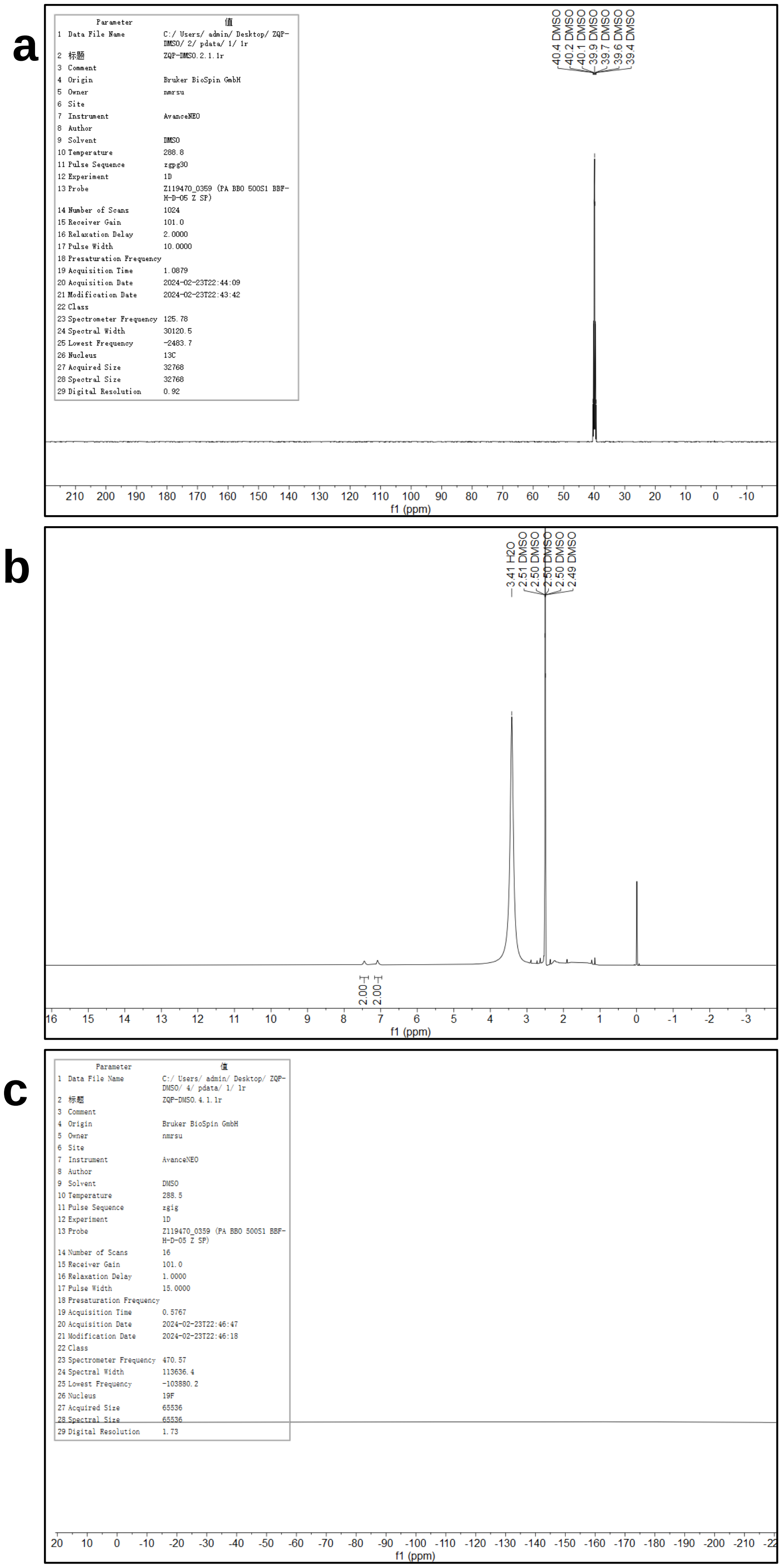

Figure S13. NMR data of oP-CDs when DMSO was used as the solvent. a, Element C. b, Element H. c, Element F.

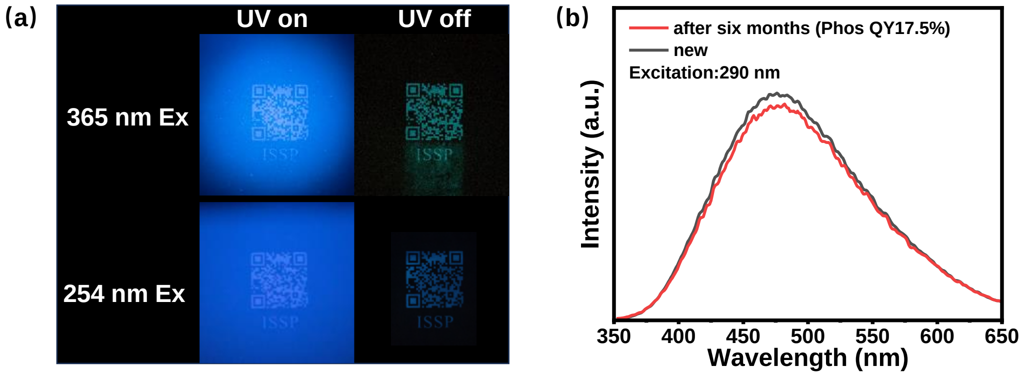

Figure S14. a.Photographs of oP-CDs@B_2O_3 after six months at constant room temperature 20 °C, excited with 254 nm & 365 nm UV lamp, and after removing UV. b. Comparison of phosphorescence spectral curves of printing ink raw materials after six months at constant room temperature 20 °C, with newly synthesized ink raw materials.

5. Supplementary Tables

Table S1. Phosphorescence lifetime of the oP-CDs@B_2O_3

Uv ex	τ_1(s)	B_1(%)	τ_2(s)	B_2(%)	τ_{avg}(s)	Phos QY(%)
254 nm	2.40	84%	4.84	16%	3.069	13.8
290 nm	1.57	47%	2.93	53%	2.483	19.5
365 nm	1.77	24%	3.87	76%	3.615	10.2

Table S2. Comparison of the emission properties of recently reported CDs-based phosphorescence materials

materials	Phos. λ_{em}(nm)	Phos. color	τ_{avg}	Glow time	Ref
NP-CPDs	535	green	1.48 s	23 s	[1]
CDs/Na0.5Mg0.25Cl	560	Yellow to green	464 ms	4 s	[2]
P-CDs@B_2O_3	510	green	1.96 s	30 s	[3]
7HOCA/BA	500	cyan	1.74 s	26 s	[4]
N-doped CDs	540	yellow	1.51 s	12 s	[5]
a-CDs/BA	530	green	1.6 s	8 s	[6]
B-CD@SiO_2	465	blue	2.11 s	21 s	[7]
G-CD@SiO_2	500	green	1.42 s	15 s	[7]
AP-CDs	470	blue	315.73 ms	5.5 s	[8]
CNDs	520	green	1.33 s	15 s	[9]
e,s-CDs@CA	550	yellow	1.14 s	12 s	[10]
oP-CDs@B_2O_3	**470**	**blue**	**3.07 s**	**33 s**	**This work**
	**500**	**green**	**3.62 s**	**49 s**	

Table S3. Phosphorescence lifetime of the oP-CDs

**Uv ex**	**Phos QY(%)**
254 nm	9.1
290 nm	12.5
365 nm	7.2

6. References

[1] Z. F. Wang, J. Shen, J. Z. Sun, B. Xu, Z. H. Gao, X. Wang, L. T. Yan, C. F. Zhu, X. G. Meng, *J Mater Chem C* **2021**, *9*, 4847-4853.

[2] W. Shi, R. Wang, J. Liu, F. Peng, R. Tian, C. Lu, *Angewandte Chemie International Edition* **2023**, *62*, e202303063.

[3] Z. Zhou, Z. Song, J. Liu, B. Lei, J. Zhuang, X. Zhang, Y. Liu, C. Hu, *Adv. Opt. Mater.* **2022**, *10*, 2100704.

[4] S. Cui, B. Wang, Y. Zan, Z. Shen, S. Liu, W. Fang, X. Yan, Y. Li, L. Chen, *Chemical Engineering Journal* **2022**, *431*, 133373.

[5] Y. Gao, H. Zhang, S. Shuang, C. Dong, *Adv. Opt. Mater.* **2020**, *8*, 1901557.

[6] W. Li, W. Zhou, Z. Zhou, H. Zhang, X. Zhang, J. Zhuang, Y. Liu, B. Lei, C. Hu, *Angewandte Chemie International Edition* **2019**, *58*, 7278-7283.

[7] Y. Zhang, M. Li, S. Lu, *Small* **2022**, *19*, 2206080.

[8] H. Shi, Y. Wu, J. Xu, C. Zhou, H. Xu, W. Ye, Y. Yin, Z. Wang, R. Su, Z. An, H. Shi, *Chemical Engineering Journal* **2023**, *476*, 146524.

[9] Y.-C. Liang, S.-S. Gou, K.-K. Liu, W.-J. Wu, C.-Z. Guo, S.-Y. Lu, J.-H. Zang, X.-Y. Wu, Q. Lou, L. Dong, Y.-F. Gao, C.-X. Shan, *Nano Today* **2020**, *34*, 100900.

[10] Y. Zheng, Q. Zhou, Y. Yang, X. Chen, C. Wang, X. Zheng, L. Gao, C. Yang, *Small* **2022**, *18*, 2201223.

7. Author Contributions

Qipeng Zhang: data curation, formal analysis, investigation, validation, writing of original draft. Lead of contribution.

Shihao Xu: data curation, investigation. Supporting of contribution.

Lanpeng Zhang: data curation, investigation. Supporting of contribution.

Liang Yang*: funding acquisition, investigation, project administration, validation, writing of original draft.

Changlong Jiang*: funding acquisition, investigation, project administration, validation, writing of original draft.
